# A Collection of Melon (*Cucumis melo*) Fruit Cultivars with Varied Skin Appearances Provide Insight to the Contribution of Suberin in Periderm Formation and Reticulation

**DOI:** 10.3390/plants11101336

**Published:** 2022-05-18

**Authors:** Ekaterina Manasherova, Hagai Cohen

**Affiliations:** Volcani Center, Department of Vegetable and Field Crops, Institute of Plant Sciences, Agricultural Research Organization (ARO), Rishon LeZion 7505101, Israel

**Keywords:** melon (*Cucumis melo*), fruit skin reticulation, suberin pathway, lignin, gas chromatography-mass spectrometry (GC-MS), very long chain fatty acids (VLCFAs)

## Abstract

At times of fruit skin failure, reticulation made of a wound-periderm is formed below the cracked skin in order to seal the damaged tissue. Preceding investigations shed light on the mechanisms underlying the formation of fruit skin reticulation, demonstrating that the walls of periderm cells are heavily suberized and lignified. However, the relative contribution of the suberin pathway to these processes, as well as the association between suberin contents in the periderm tissue and reticulation degree, are largely unknown. To strengthen our understanding on these important physiological and agricultural aspects, we comparatively profiled skin tissues of a collection of smooth- and reticulated-skin melon (*Cucumis melo*) cultivars for suberin monomer composition via gas chromatography-mass spectrometry (GC-MS). This metabolite profiling approach accompanied by statistical tools highlighted the fundamental chemical differences between the skin of smooth fruit made of a typical cuticle, to the skin of reticulated fruit made of large amounts of archetypal suberin building blocks including hydroxycinnamic acids, very long chain fatty acids, fatty alcohols, α-hydroxyacids, ω-hydroxyacids, and α,ω-diacids. Next, using image analysis we generated ‘reticulation maps’ and calculated the relative densities of reticulation. We then performed correlation assays in order to monitor suberin monomers that specifically correlate well with reticulation degree. Nonetheless, total suberin contents and most suberin building blocks did not show high correlations with reticulation degree, further suggesting that additional factors are likely to influence and regulate these processes. Altogether, the data provided vital information regarding the relative contribution of the suberin pathway to periderm formation and skin reticulation.

## 1. Introduction

Melon (*Cucumis melo*) is an important agricultural crop belonging to the Cucurbitaceous family. Fruit produced by wild melon varieties are typically small with very little edible mesocarp tissue and their skin surfaces are smooth and made of a thick cuticle [1]. On the other hand, fruit of domesticated melon varieties are much larger and their skin surfaces display a large phenotypic diversity from fruit with a smooth skin appearance to fruit with different types of reticulation decorations. The unique phenomenon of skin reticulation in melon fruit fascinated researchers as early in the 1950s that described it as “a tissue originating from cracks appearing on the fruit surface” [2]. Later investigations demonstrated that during the development of reticulated-skin melon fruit, small fissures or microcracks emerge on the skin surface. These are turning longer and wider, becoming macrocracks and occupying larger areas of the skin surface, and eventually moving towards fruit maturity, the fissures interconnect to from a complete reticulated structure [3,4]. Histological studies showed that cells below these fissures start to multiply rapidly, forming a typical wound-periderm made of a mass of cells with heavily suberized and lignified walls [5]. The establishment of a wound-periderm below the cracked skin holds important roles in facilitating a relatively normal development of the fruit even under circumstances of skin failure. These include the maintenance of fruit firmness but at the same time allow its elasticity, which is necessary during the expansion of fruit volume [6,7,8], and securing turgor pressure and waterproofing capacity of epidermal and pericarp cells below the fruit skin tissues [9]. As most of the studies on melon fruit skin reticulation that drew conclusions on this process were based on simple microscopical observations, we recently initiated a multi-omics investigation in order to untangle the molecular and biochemical features associated with melon fruit skin reticulation. Using high-resolution microscopy accompanied by several metabolite profiling approaches, we were able to highlight structural attributes, chemical compositions, and gene expression profiles that tightly associate with melon fruit skin reticulation [10]. More recently, we accomplished a corresponding project where we examined fruit of the Sikkim cucumber (*Cucumis sativus* var. *sikkimensis*) whose skin undergoes massive reticulation during development. Engagement of advanced microscopy, metabolomics, and proteomics enabled the characterization of structural, metabolic, and proteomic changes that occur in the fruit skin at early and late developmental stages [11].

It is clear from the above studies, as well as from additional previous evidence, that the involvement of the suberin biosynthetic pathway and the introduction of suberin building blocks in the walls of wound-periderm cells are crucial to the formation of reticulation on the skin surface. Nevertheless, it is not clear whether there is a direct link between the amount of suberin content and/or the levels of specific suberin monomers to the degree of fruit skin reticulation. To further tackle this notion, we comparatively profiled skin tissues of a collection of 28 melon cultivars with smooth- or reticulated-skin appearances for suberin monomer composition via gas chromatography-mass spectrometry (GC-MS). We employed varied statistical tools to highlight fundamental chemical differences between the skin tissues of smooth- and reticulated fruit cultivars. Simultaneously, skin reticulation densities were calculated for all investigated reticulated-skin fruit cultivars using image analyses, and correlation assays were performed in order to monitor suberin monomers that specifically correlate with reticulation degree. Altogether, the data provided vital information regarding the relative contribution of the suberin pathway to periderm formation and skin reticulation.

## 2. Results

### 2.1. The Utilization of Melon (Cucumis melo) Fruit Cultivars with Varied Skin Appearances to Study the Contribution of Suberin in Periderm Formation and Reticulation

**Figure 1:**
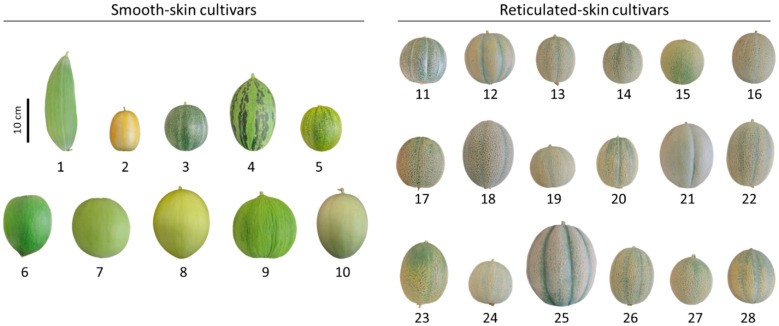
The collection of melon (*Cucumis melo*) cultivars with phenotypes of smooth- and reticulated-skin that was investigated in the current study. The numbers below each melon image correspond to those appearing in Table 1.

In this study, we used a commercially available collection of smooth-skin melons (10 cultivars: Banana, Early Silver Line, Petit Gris de Renes, Piel de Sapo, Collective Farm Woman, Crenshaw, Green Flesh Honeydew, Juane Canary, Casaba Golden Beauty, and Tam Dew) and reticulated-skin melons (18 cultivars: Emerald Green Gem, Golden Delicious, Green Nutmeg, Jenny Lind, Minnesota Midget, Planter’s Jumbo, Hale’s Best 36, Hale’s Best Jumbo, Honey Rock, Rocky Ford Green Flesh, Schoon’s Hardshell, Sierra Gold, Edisto 47 Cantaloupe, Granite State, Iroquois, PMR 45, Top Mark, and Tuscany) (Table 1). Dry seeds from all 28 melon cultivars were ordered from the Sustainable Seed Company (www.sustainableseed.com, accessed on 8 May 2022) and grown in Israel (for growth conditions please see Material and Methods). Fruit produced by these cultivars varied in skin color, fruit size, and maturity indexes, whereas reticulated fruit displayed unalike degrees of skin reticulation (Figure 1). Thus, the collection serves as a suitable genetic platform to deepen our knowledge of the chemical differences between smooth- and reticulated-skin melon fruit, and to elucidate a link between reticulation degree to these chemical attributes.

### 2.2. GC-MS Polyester Analysis Highlights Fundamental Chemical Differences between Skin Tissues of Smooth- and Reticulated-Skin Melon Fruit

**Figure 2:**
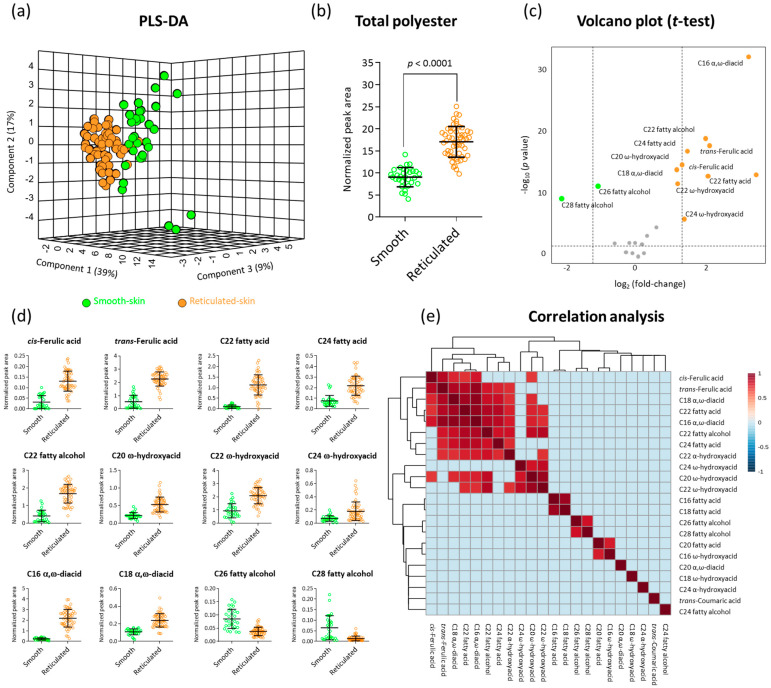
GC-MS polyester analysis highlights fundamental chemical differences between skin tissues of smooth- and reticulated-skin melon fruit. (**a**) A 3D partial least square-discriminant analysis (PLS-DA) displaying separation between skin tissues of smooth- and reticulated-skin melon fruit based on the levels of 22 positively annotated metabolites following GC-MS analysis. The variances explained by each of the first three components are displayed in parentheses. (**b**) Total polyester contents in skin tissues of smooth- and reticulated-skin melon fruit based on the levels of 22 positively annotated metabolites following GC-MS analysis. The y-axis represents the total peak area following normalization to the C32 internal standard. (**c**) A volcano plot showing the significant metabolites that were higher in reticulated-skin tissues (orange dots), higher in smooth-skin tissues (green dots), and those that were not statistically different between the two tissues (grey dots) following a *t*-test analysis of *p* value < 0.05. (**d**) Scatter dot plots repressing the levels of 12 significantly-changed metabolites between skin tissues of smooth- and reticulated-skin melon fruit following a *t*-test analysis as appear in (**c**). All 12 metabolites exhibited significantly different levels between skin samples in *p* values < 0.0001. Each dot represents individual biological replicates of different smooth- and reticulated-skin samples, while black lines represent means ± SD. (**e**) Correlation analysis between all the 22 GC-MS-based positively-annotated metabolites according to their abundances in smooth- and reticulated-skin skin samples. Correlation was calculated according to Spearman rank method. Color scale displays correlations within the range of −1 to 1. A correlation cutoff of 0.7 was applied to show only strongly-correlated metabolites.

We carried out a comparative GC-MS profiling of polyester chemical compositions in skin tissues isolated from the fruit of all investigated smooth- and reticulated-skin cultivars. Based on public and in-house mass libraries, we were able to positively annotate 22 typical cutin and suberin monomers belonging to the biochemical classes of hydroxycinnamic acids, fatty acids, fatty alcohols, α-hydroxyacids, ω-hydroxyacids, and α,ω-diacids. Plotting skin samples onto a partial least square-discriminant analysis (PLS-DA) based on their profile of these 22 metabolites pointed at a clear separation between skin of smooth and reticulated fruit (Figure 2a). The first and second principal components explained 56% of the variance among skin samples, while the third principal component contributed an additional 9% (Figure 2a).

Calculations of total polyester contents in skin samples demonstrated that reticulated-skin accumulated almost 2-fold more polyester content compared to smooth-skin (Figure 2b). In order to isolate the metabolites that have levels that are significantly different between the two types of skin, a *t*-test statistical analysis (*p* value < 0.05) was employed yielding 10 metabolites that exhibited higher abundances in reticulated-skin and 2 metabolites that were higher in smooth-skin out of the 22 annotated metabolites, as presented by a volcano plot (Figure 2c).

We then examined the exact levels of each of these metabolites in smooth- and reticulated-skin samples. The 10 metabolites that had significantly higher abundances in reticulated-skin were *cis*-Ferulic acid, *trans*-Ferulic acid, C22 fatty acid, C24 fatty acid, C22 fatty alcohol, C20 ω-hydroxyacid, C22 ω-hydroxyacid, C24 ω-hydroxyacid, C16 α,ω-diacid, and C16 α,ω-diacid, exhibiting higher levels ranging between 2- to 11-fold changes (Figure 2d). On the other hand, the levels of C26 and C28 fatty alcohols were 2- and 5-fold higher in smooth-skin compared to reticulated-skin (Figure 2d).

Finally, we performed a correlation analysis where we monitored metabolites that exhibited strong correlation with other metabolites. The analysis highlighted *cis*-Ferulic acids, *trans*-Ferulic acid, C18 α,ω-diacid, C22 fatty acid, C24 fatty alcohol, C24 α-hydroxyacid, C20 ω-hydroxyacid, and C22 ω-hydroxyacid that exhibited strong correlations with each other (Figure 2e). Interestingly, all of these metabolites were among those that highly accumulated in reticulated-skin fruit skin. On the other side, the two fatty alcohols, C26 and C28, which were significantly lower in reticulated-skin as compared with smooth-skin, were highly correlated with each other (Figure 2e).

### 2.3. Image Analysis and Reticulation Density Calculations in Skin of Reticulated Fruit

**Figure 3:**
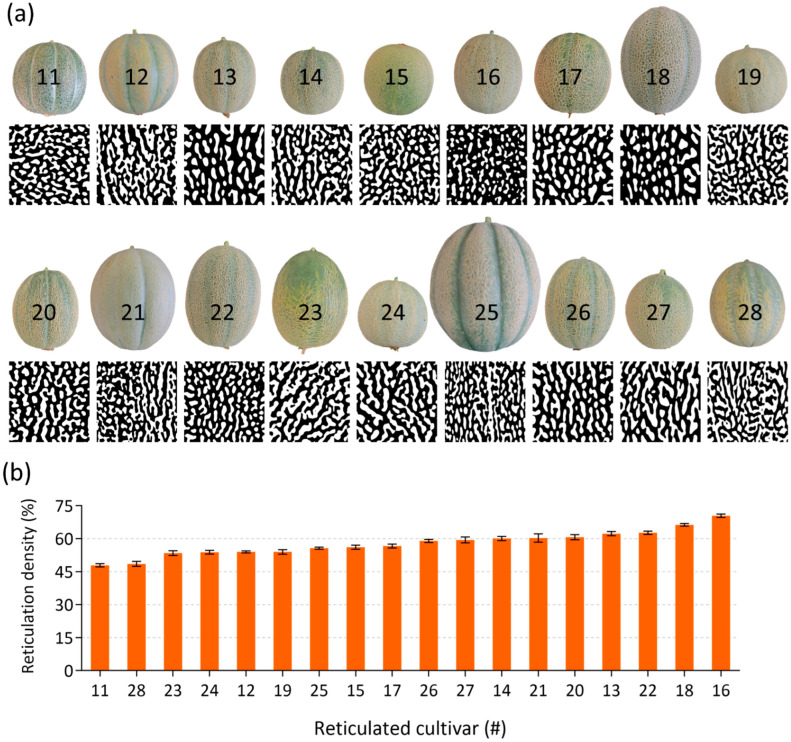
Image analysis and reticulation density calculations in skin of reticulated fruit. (**a**) Representative reticulation maps of skin belonging to the 18 reticulated-skin fruit cultivars investigated in the current study as appears in Table 1. (**b**) Calculations of reticulation densities in the 18 reticulated-skin fruit cultivars. Bar graphs represent means ± SD. *n* = 8.

Skin reticulation patterning seemed to differ between the 18 reticulated-skin fruits. An additional aim of our research was to examine the relative contribution of suberin biosynthesis pathway and/or specific suberin monomers to reticulation density. We were therefore interested to quantify the degree of reticulation in each of these reticulated cultivars, i.e., calculating the relative surface area of fruit skin that is covered by reticulation structures. High-resolution images were taken from fruit of all reticulated-skin cultivars, which were then transformed into black and white images to circumvent inaccurate identification of reticulated structure that arise from natural differences in skin color. The later images were used to determine the overall reticulation density from each reticulated-skin cultivar (Figure 3a). Reticulation densities ranged from the lowest 48% in reticulated-skin cultivar 11 (Emerald Green Gem) to the highest 70% detected in the reticulated-skin cultivar 16 (Planter’s Jumbo) (Figure 3b).

### 2.4. Correlation Analyses between Suberin Monomers and Reticulation Density in Reticulated-Skin Melon Cultivars

**Figure 4:**
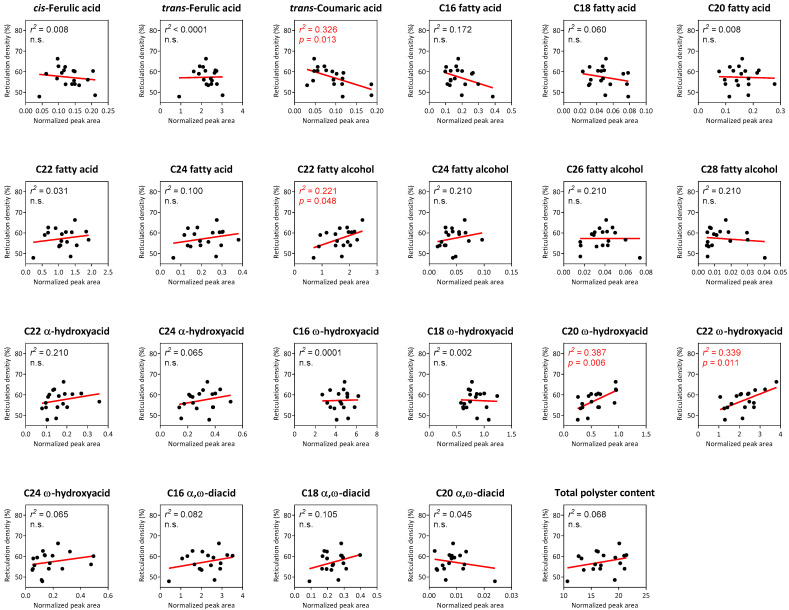
Correlation analyses between suberin monomers and reticulation density in reticulated-skin melon cultivars. Graph axes represent reticulation density (%) vs. the normalized peak area of each suberin monomer identified following GC-MS analyses. Black dots represent 18 reticulated-skin cultivars, while red lines represent the calculated linear regression fit for each suberin monomer. Calculated *r*^2^ and *p* values are presented in the top left corner of every graph, where n.s. equals non-significant.

Lastly, we obtained the suberin monomer profiles for all reticulated-skin fruit in addition to reticulation densities for each of these cultivars. This allowed us to compute the linear regressions between total polyester contents and individual identified monomers to calculated reticulation densities. Surprisingly, relatively low regressions were found between the variables, and in fact among all them only four individual metabolites displayed either negative or positive significant linear regressions with reticulation density. These included *trans*-Coumaric acid that exhibited a negative regression (*r*^2^ = 0.326; *p* value = 0.013), and C22 fatty alcohol (*r*^2^ = 0.221; *p* value = 0.048), C20 ω-hydroxyacid (*r*^2^ = 0.387; *p* value = 0.006) and C22 ω-hydroxyacid (*r*^2^ = 0.339; *p* value = 0.011), which displayed positive regression with reticulation density (Figure 4). Additionally, total polyester contents did not exhibit a significant regression with reticulation density (Figure 4).

## 3. Discussion

In the current study we took advantage of a diverse collection of melon cultivar with either smooth or reticulated skin. By performing a comparative GC-MS metabolite profiling of polyester contents and compositions, we were able to show that the establishment of a wound-periderm tissue and reticulation upon the fruit surface are tightly associated with the accumulation of typical suberin building blocks. These results are in line with our previous studies untangling the molecular mechanisms involved in fruit skin reticulation in melon [10]. However, the involvement of the suberin biosynthesis pathway in periderm tissue formation was also reported previously in other fruit species like the Sikkim cucumber that is also decorated by a thick reticulated corky skin [11], as well as in apple (*Malus* × *domestica*) and pear (*Pyrus communis*) fruit varieties that undergo russeting—another skin phenomenon that occurs due to macrocracking of the fruit surface [12,13], in periderm tissues of potato tubers [14], tomato [15] and kiwifruit [16].

The skin of fleshy fruit is made of a typical thick cuticle made predominantly of the cutin polyester—a mixture of C16 and C18 fatty acid derivatives with hydroxy and epoxy groups [17,18]. Upon skin failure, the fruit generates a wound-periderm underneath the cracked skin that is usually made of suberized and lignified cells, and therefore the chemistry of the skin is significantly modified. Unlike the cutin polyester, suberin is a highly aromatic-containing hydroxycinnamic acid that originates from the core phenylpropanoid pathway. Indeed, our assays showed that all reticulated-skin fruit accumulated very large amounts of *cis*- and *trans*-Ferulic acids as compared to the smooth-skin fruit. While these two metabolites were tightly correlated with some other suberin-building blocks apparently forming a sort of core subset of periderm suberin metabolites, they did not show any significant linear regression with reticulation densities. On the other hand, *trans*-Coumaric acid that is typically associated with the cutin polyester displayed a significant linear regression with reticulation density, hence suggesting that higher reticulation densities accompany reductions in some cuticle components. Truly, reductions in hydroxycinnamic acids associated with the cuticle such as coumaric and caffeic acids, were previously reported in melon [10], cucumber [11], and apple [19].

Apart from its aromatic domain, suberin was also composed of a diverse aliphatic domain made of fatty acid derivatives with chain lengths longer then C20, including fatty alcohols, ω-hydroxyacids, α-hydroxyacids, and α,ω-diacids [20]. Herein, skin tissues from all reticulated fruit accumulated various metabolites belonging to these biochemical classes that were either extremely low or virtually absent in skin tissues belonging to smooth-skin fruit. Among these we found that C22 and C24 fatty acids; C22 fatty alcohol; C20, C22, and C24 ω-hydroxyacids; and C16 and C18 α,ω-diacids were the most predominant and significant ones that accumulated to large amounts in the periderm tissues of reticulated-skin fruit. Remarkably, the levels of most of these metabolites were also highly correlated with each other, further strengthening the possibility that they constitute a core metabolic fingerprint of a suberized periderm tissue in melon. Three of these metabolites (C22 fatty alcohol, and C20 and C22 ω-hydroxyacids) also displayed significant positive linear regressions with reticulation densities, suggesting that they can serve as metabolic markers for fruit skin reticulation in melon varieties and likely in other species of reticulated or russeted fleshy fruit.

All in all, the data presented here provide an additional line of evidence for the important role of the suberin biosynthetic pathway during the development of wound-periderm and reticulation. At the same time, total suberin contents and most suberin building blocks did not exhibit high correlations with reticulation degree, implicating that additional factors are likely to influence and regulate these processes apart from suberin.

## 4. Materials and Methods

### 4.1. Plant Material and Cultivation Conditions

Dry melon (*Cucumis melo*) seeds of cultivars with smooth-skin (10 cultivars) or reticulated-skin (18 cultivars) were ordered from the Sustainable Seed Company (www.sustainableseed.com, accessed on 8 May 2022) and grown in a net house in Rehovot, Israel, under natural light and environmental conditions. Plants were periodically fertilized with a 20:20:20 N:P:K fertilizer. Fruit at maturity developmental stage were harvested from five independent plants of each cultivar for further analyses. Full names, skin characteristics, and days to fruit maturity are listed in Table 1. Skin tissues were manually dissected from freshly-harvested fruit to avoid any postharvest changes in skin structure and/or polymer chemistry, and immediately treated for further GC-MS assays. In addition, high-resolution images of fruit skin were taken for further image analyses and reticulation density calculations.

### 4.2. Polyester Analysis via GC-MS

Skin polyester analysis was performed according to previous GC-MS-based protocols [10,11]. Skin discs of 1 cm in diameter were excited from freshly-harvested fruit from all 28 melon cultivars using a stainless-steel cork borer. Skin discs were then delipidated for 2 consecutive weeks in a GC-MS purity grade methanol:chloroform buffer (*v*/*v*), dehydrated overnight in a hood, and inserted for 72 h to a desiccator containing activated silica-gel beads to allow full dehydration. Delipidated fully-dried skin discs were then trans-esterified using the addition of 4 mL of boron trifluoride:methanol (Sigma-Aldrich; 99.8%) and incubation for 16 h at 70 °C oven. The interna standard of *n*-Dotriacontane (C32 alkane) was added into all trans-esterified after cooling down followed by vigorous vortex. The trans-esterification process was terminated by the addition of saturated NaHCO_3_/water. Four mL of chloroform was added into the samples to extract all available cutin and/or suberin monomers. Phase separation was achieved via the addition of 1 mL of GC-MS purity grade water and vigorous vortex. The upper polar phase was gently removed using a glass pipet, and clean chloroform extracts were fully dried by the addition of anhydrous NaSO_4_. Dried chloroform extracts were transferred into 2 mL reaction vials and fully evaporated under a nitrogen stream. Prior to GC-MS running, both chloroform extracts containing cutin, suberin, or epicuticular waxes were resuspended in 100 µL of chloroform, derivatized with 20 µL of pyridine (Sigma-Aldrich, St. Louis, MO, USA; 99.8%, anhydrous), and 20 µL of *N*,*O*-bis(trimethylsilyl)trifluoroacetamide (Sigma-Aldrich, St. Louis, MO, USA; GC grade) at 70 °C for 1 h, and transferred into GC-MS vials. A sample volume of 1 µL was injected in splitless mode on a GC-MS system (Agilent 7693A Liquid Auto injector, 8860 gas chromatograph, and 5977B mass spectrometer). GC was performed (HP-5MS UI column; 30 m length, 0.250 mm diameter, and 0.25 µm film thickness; Agilent J&W GC Columns) with an injection temperature of 270 °C, the interface set to 250 °C, and the ion source to 200 °C. Helium was used as the carrier gas at a constant flow rate of 1.2 mL min^−1^. The temperature program was 0.5 min isothermal at 70 °C, followed by a 30 °C min^−1^ oven temperature ramp to 210 °C and a 5 °C min^−1^ ramp to 330 °C, then kept constant for 21 min. Mass spectra were recorded with an *m/z* 40 to 850 scanning range. Chromatograms and mass spectra were evaluated using the MSD ChemStation software (Agilent Technologies, Santa Clara, CA, USA). Integrated peaks of mass fragments were normalized for the respective C32 alkane internal standard signal. For identification, the corresponding mass spectra and retention time indices were compared with the NIST20 library as well as in-house spectral libraries.

### 4.3. Image Analysis and Calculations of Reticulation Densities

Reticulation densities in all 18 reticulated-skin cultivars were based on high-resolution skin surface images obtained from eight independent fruit for each reticulated-skin cultivar. First, an in-house script was generated to transform images into black and white to circumvent inaccurate identification of a reticulated structure that arises from natural differences in skin color. Then, reticulation maps were generated for every image. The in-house script was generated using the R software (https://www.r-project.org/, accessed on 8 May 2022), which was then implemented into the ImageJ software (https://imagej.net/, accessed on 8 May 2022) for final determination of reticulation densities.

### 4.4. Statistical Analyses

All statistical analyses and generation of graphs were performed using the tools embedded in GraphPad Prism v8.0.1, a versatile statistics tool purpose-built for scientists (https://www.graphpad.com/, accessed on 8 May 2022), or in MetaboAnalyst v5.0, a comprehensive tool suite for metabolomic data analysis (http://metaboanalyst.ca/, accessed on 8 May 2022; [21]). Significance was calculated according to the two-tailed *t*-test statistical method of *p*-value < 0.05. The number of biological replicates is mentioned in the corresponding figure legend of each experiment. PLS-DA, volcano plot (*t*-test of *p* value < 0.05), and metabolite correlation (according to the Spearman rank method with a correlation cutoff of 0.7) were performed in MetaboAnalyst v5.0 after GC-MS data were normalized according to the peak area of the C32 internal standard, log_10_-transformed, and scaled according to the auto-scaling method (mean-centered and divided by the standard deviation of each variable). Linear regression graphs delineating the association between total polyester content, or the levels of each of the annotated 22 monomers, to calculated reticulation densities in reticulated-skin fruit, were generated using the GraphPad Prism v8.0.1 including *r*^2^ and *p* values for association.

## 5. Conclusions

In the current study we took advantage of a large collection of melon fruit varieties with either smooth- or reticulated-skin appearances. This allowed us to comparatively profile them for cutin and suberin building blocks in their skin polyester fractions. In accordance with our previous observations, the skin of smooth fruit is made from typical cuticle components, while the induction of a wound-periderm in the reticulated fruit is accompanied with the accumulation of a large amount of archetypical suberin monomers. Among these metabolites, ferulic acid dominated the aromatic moiety of suberin, where C20–C24 fatty acids, alcohols, and ω-hydroxyacids, and C16 and C18 α,ω-diacids represent the aliphatic domain. The levels of all these metabolites where highly correlated, suggesting that this metabolite subset might represent the core components of suberin in the periderm tissue of reticulated melon fruit. Despite the crucial contribution of the suberin biosynthetic pathway to wound periderm formation, it showed only minor correlation with reticulation densities. Hence, we postulate that even though the role of the suberized wound periderm is to seal the cracked skin, the buildup of reticulation on top of the skin depends on many other determinants apart from the accumulation of suberin. Additional biochemical and molecular studies are required in order to better understand the relationships between these factors. Furthermore, tackling these questions in other reticulated or russeted fruit species could potentially provide valuable insight into the underlying mechanisms that govern fruit skin reticulation, suberin, and their interconnection.

## Figures and Tables

**Table 1:** Full names, skin characteristics, and days to mature fruit of smooth- and reticulated-skin melon (*Cucumis melo*) cultivars investigated in the current study. Cultivar numbers correspond to those that appear in Figure 1.

Cultivar Number	Cultivar Name	Skin Characteristics	Days to Mature Fruit
1	Banana	Smooth	90
2	Early Silver Line	Smooth	76
3	Petit Gris de Rennes	Smooth	85
4	Piel de Sapo	Smooth	103
5	Collective Farm Woman	Smooth	83
6	Crenshaw	Smooth	110
7	Green Flesh Honeydew	Smooth	105
8	Juane Canary	Smooth	82
9	Casaba Golden Beauty	Smooth	100
10	Tam Dew	Smooth	100
11	Emerald Green Gem	Reticulated	78
12	Golden Delicious	Reticulated	83
13	Green Nutmeg	Reticulated	85
14	Jenny Lind	Reticulated	79
15	Minnesota Midget	Reticulated	70
16	Planter’s Jumbo	Reticulated	89
17	Hale’s Best 36	Reticulated	85
18	Hale’s Best Jumbo	Reticulated	85
19	Honey Rock	Reticulated	100
20	Rocky Ford Green Flesh	Reticulated	89
21	Schoon’s Hardshell	Reticulated	88
22	Sierra Gold	Reticulated	90
23	Edisto 47 Cantaloupe	Reticulated	86
24	Granite State	Reticulated	83
25	Iroquois	Reticulated	75
26	PMR 45	Reticulated	90
27	Top Mark	Reticulated	90
28	Tuscany	Reticulated	90

## Data Availability

Not applicable.

## References

[1] Monforte A.J., Diaz A., Cano-Delgado A., van der Knaap E. (2014). The genetic basis of fruit morphology in horticultural crops: Lessons from tomato and melon. J. Exp. Bot..

[2] Meissner F. (1952). Die Korkbildung der Fruechte von Aesculusund Cucumis-Arten. Osstereichsche Bot. Z..

[3] Cutler E.G. (1969). Plant Anatomy: Experiment and Interpretation.

[4] Webster B.D., Craig M.E. (1976). Net morphogenesis and characteristics of the surface of melon fruit. J. Am. Soc. Hortic. Sci..

[5] Keren-Keiserman A., Tanami Z., Shoseyov O., Ginzberg I. (2004). Rind characteristics associated with melon (*Cucumis melo*) netting: Comperative study with smoothed-rind vareities. J. Hortic. Sci. Biotechnol..

[6] Rose J.K.C., Hadfield K.A., Labavitch J.M., Bennett A.B. (1998). Temporal sequence of cell wall disassembly in rapidly ripening melon fruit. Plant Physiol..

[7] Dos-Santos N., Jimenez-Araujo A., Rodriguez-Arcos R., Fernandez-Trujillo J.P. (2011). Cell wall polysaccharides of near-isogenic lines of melon (*Cucumis melo* L.) and their inbred parentals which show differential flesh firmness or physiological behavior. J. Agric. Food Chem..

[8] Puthmee T., Takahashi K., Sugawara M., Kawamata R., Motomura Y., Nishizawa T., Aikawa T., Kumpoun W. (2013). The role of net development as a barrier to moisture loss in netted melon fruit (*Cucumis melo* L.). HortScience.

[9] Saladié M., Matas A.J., Isaacson T., Jenks M.A., Goodwin S.M., Niklas K.J., Xiaolin R., Labavitch J.M., Shackel K.A., Fernie A.R. (2007). A re-evaluation of the key factors that influence tomato fruit softening and integrity. Plant Physiol..

[10] Cohen H., Dong Y., Szymanski J., Lashbrooke J., Meir S., Almekias-Siegl E., Zeisler-Diehl V.V., Schreiber L., Aharoni A. (2019). A multilevel study of melon fruit reticulation provides insight into skin ligno-suberizattion hallmarks. Plant Physiol..

[11] Arya G.C., Dong Y., Heinig U., Shahf N., Kazachkova Y., Aviv-Sharon E., Nomberg G., Marinov O., Manasherova E., Aharoni A. (2022). The metabolic and proteomic repertoires of periderm tissue in skin of the reticulated Sikkim cucumber fruit. Horticul. Res..

[12] Khanal B.P., Grimm E., Knoche M. (2013). Russeting in apple and pear: A plastic periderm replaces a stiff cuticle. AoB Plants.

[13] Lashbrooke J., Aharoni A., Costa F. (2015). Genome investigation suggests MdSHN3, an APETALA2-domain transcription factor gene, to be a positive regulator of apple fruit cuticle formation and an inhibitor of russet development. J. Exp. Bot..

[14] Graca J., Pereira H. (2000). Suberin structure in potato periderm: Glycerol, long-chain monomers, and glyceryl and feruloyl dimers. J. Agric. Food Chem..

[15] Tao X., Mao L., Li J., Chen J., Lu W., Huang S. (2016). Abscisic acid mediates wound-healing in harvested tomato fruit. Postharvest Biol. Technol..

[16] Han X., Mao L., Wei X., Lu W. (2017). Stimulatory involvement of abscisic acid in wound suberization of postharvest kiwifruit. Sci. Hortic..

[17] Pollard M., Beisson F., Li Y., Ohlrogge J.B. (2008). Building Lipid Barriers: Biosynthesis of Cutin and Suberin. Trends Plant Sci..

[18] Arya G.C., Sarkar S., Manasherova E., Aharoni A., Cohen H. (2021). The Plant Cuticle: An Ancient Guardian Barrier Set Against Long-Standing Rivals. Front. Plant Sci..

[19] Legay S., Guerriero G., Deleruelle A., Lateur M., Evers D., André C.M., Hausman J.F. (2015). Apple russeting as seen through the RNA-seq lens: Strong alterations in the exocarp cell wall. Plant Mol. Biol..

[20] Nomberg G., Marinov O., Arya G.C., Manasherova E., Cohen H. (2022). The key enzymes in the suberin biosynthetic pathway in plants: An update. Plants.

[21] Pang Z., Chong J., Zhou G., de Lima Morais D.A., Chang L., Barrette M., Gauthier C., Jacques P.É., Li S., Xia J. (2021). MetaboAnalyst 5.0: Narrowing the gap between raw spectra and functional insights. Nucleic Acids Res..

